# The burden of congenital hyperinsulinism in the United Kingdom: a cost of illness study

**DOI:** 10.1186/s13023-018-0867-6

**Published:** 2018-07-20

**Authors:** Sana Eljamel, Annabel Griffiths, Jenni Evans, Indraneel Banerjee, Khalid Hussain, Richard Thompson

**Affiliations:** 1 Costello Medical, Cambridge, UK; 2 Royal Manchester Children’s Hospital, Manchester, UK; 3 Great Ormond Street Hospital for Children NHS Trust, London, UK; 4 Findacure, 66 Devonshire Road, Cambridge, CB1 2BL UK

**Keywords:** Congenital hyperinsulinism, Persistent hyperinsulinaemic hypoglycaemia of infancy, Cost of illness, Economic burden of disease

## Abstract

**Background:**

Congenital hyperinsulinism (CHI) is a rare, genetic disease which causes persistent hypoglycaemia, typically in new-borns. Patients with the diffuse disease variant often require near-total surgical removal of the pancreas, causing insulin-dependent diabetes mellitus (IDDM). The CHI economic burden is currently unknown. This study aimed to estimate the annual cost of illness (COI) of CHI patients in the UK from a service provider perspective (National Health Service, NHS and Personal Social Services), and to explore cost distribution within the patient population.

**Methods:**

The model was based on standard practice of two CHI centres of excellence. Model inputs were informed by a pragmatic literature review, NHS Reference Costs (2015–2016) and the British National Formulary (2017). Only direct costs to the NHS and Personal Social Services were considered. A prevalence-based approach was used and annual costs incurred at all ages were calculated. A deterministic sensitivity analysis (DSA; run at 10%) identified major cost drivers.

**Results:**

The COI of CHI patients to the NHS was £3,408,398.59 annually and average cost per patient was £2124.95. Cost distribution was skewed among CHI patients, with 5.9% of patients (95 patients in their first year of life) contributing to 61.8% (£2,105,491.07) of total costs. DSA results identified lack of response to first-line therapy and IDDM development post surgery (and associated healthcare costs) as major cost drivers.

**Conclusions:**

Despite its rare disease status, estimated annual costs of CHI to the NHS were substantial. Development and management of post-surgical IDDM as a major cost driver highlights the need for effective treatments to mitigate such consequences and costs.

**Electronic supplementary material:**

The online version of this article (10.1186/s13023-018-0867-6) contains supplementary material, which is available to authorized users.

## Background

Congenital hyperinsulinism (CHI) is a rare, genetic disease characterised by excessive and unregulated insulin secretion from the β-cells of the pancreas, resulting in persistent and severe hypoglycaemia (low blood glucose) [1, 2]. The condition usually presents at birth and causes symptoms such as loose or floppy muscles, shakiness, poor feeding, and seizures [1]. CHI has an estimated global prevalence of 1/50,000, [3] with approximately 2170 people living with the disease in the UK [4]. Clinical experts in CHI estimate that approximately 95 infants are born with the disease in the UK each year [Expert opinion, Great Ormond Street Hospital; GOSH, 2015 and Northern Congenital Hyperinsulinism Service, NORCHI, 2017]. Of these, up to a third may experience impaired neurodevelopment as a result of hypoglycaemia [5, 6]. Delays to treatment have been found to have a deleterious effect on neurodevelopmental outcomes. Therefore, prompt diagnosis and treatment are essential to avoid permanent brain damage and long-term consequences of impaired neurodevelopment such as epilepsy, psychomotor impairment, learning difficulties, and further cerebral sequelae [1, 6–8].

**Fig. 1:**
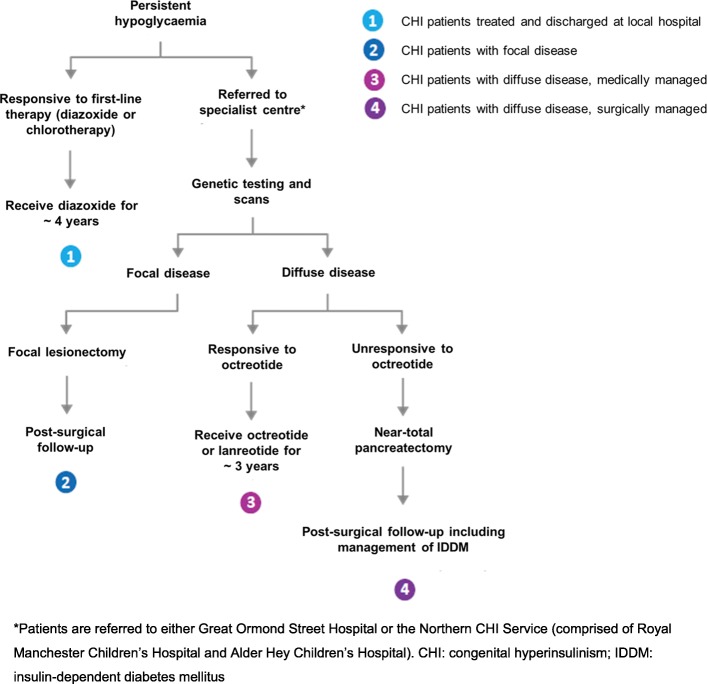
Treatment pathway depicting the management of CHI

There are two histological subtypes of CHI: focal disease, in which a discrete and specific area of the pancreas is affected; and diffuse disease, in which β-cells distributed throughout the entire pancreas are affected [1, 2]. The primary aim of treatment in either case is blood glucose stabilisation and reduction of insulin levels in order to prevent neurological complications [9]. Acute treatment may include parenteral glucose infusion, frequent feeding, and administration of glucagon [10]. Further treatment of CHI is more complex as the appropriate treatment approach varies according to the histology of the disease [11, 12]. The standard treatment pathway for the management of CHI in the UK is outlined below and in Fig. 1.

Diazoxide and chlorothiazide are usually the first lines of treatment considered for infants who present with persistent hypoglycaemia. Patients who respond to these treatments are treated at, and discharged from, their local hospital. They may continue to receive diazoxide until they experience spontaneous clinical improvement, usually after approximately 4 years [3, 13]. Patients who are unresponsive to these first-line drugs are then referred to a specialist centre for genetic tests and scans in order to determine whether the infant has focal or diffuse disease.

Patients with focal disease who are unresponsive to first-line drugs can be treated by surgical removal of the affected area of the pancreas via a focal lesionectomy, a procedure which is usually curative [10]. Patients with diffuse disease, however, are typically treated with diazoxide as first-line therapy. If diazoxide is ineffective, octreotide, a somatostatin analogue which inhibits insulin secretion, may be used as a second-line therapy [3, 14]. Response to octreotide precludes the need for further treatment; patients who respond to octreotide treatment will continue to receive octreotide until they experience spontaneous clinical improvement, usually after approximately 4 years [3, 9]. Patients with an inadequate response to octreotide usually require a near-total pancreatectomy – a surgical procedure in which approximately 90–95% of the pancreas is removed [2, 10, 11, 15]. Currently, this is the only treatment option available for these patients. Patients who have undergone this procedure have a high chance of developing life-long conditions such as insulin-dependent diabetes mellitus (IDDM) and other manifestations of pancreatic insufficiency [2, 10, 11, 15]. IDDM is also associated with a number of secondary complications such as heart disease, stroke and nerve damage [16].

Other CHI treatments are currently being investigated, including the mammalian target of rapamycin (mTOR) inhibitor sirolimus, which appears to downregulate the production of insulin from the β-cells in the pancreas and may prevent the β-cells from continually multiplying [9, 17]. Sirolimus is currently used to treat CHI in an off-label capacity by some healthcare centres (as is the case with other CHI medications). However, sirolimus was not included in this study as it is not yet widely accepted in clinical practice, although it is acknowledged that this treatment may be adopted in future clinical trials [18].

The care of patients with CHI is commissioned on a national level as a Highly Specialised Service with two major treatment centres: Great Ormond Street Hospital (GOSH), London; and the Northern Congenital Hyperinsulinism service (NORCHI), which is based at the Royal Manchester Children’s Hospital with an additional centre at the Alder Hey Children’s Hospital, Liverpool. Due to the presence of these specialist centres, the vast majority of patients in England are treated by the same clinical teams, and therefore experience a relatively similar treatment pathway.

The economic burden of CHI has not been studied to date and thus is poorly understood. To our knowledge, no cost of illness (COI) studies investigating CHI are available. Such studies would provide important clinical and economic data that can help healthcare providers and policy makers to make key decisions regarding the provision of treatments and services. The purpose of this study was to estimate the annual COI of individuals with CHI from a service provider perspective based on the treatment pathway followed by patients receiving care through the UK’s NHS, and to explore the distribution of these costs within the patient population.

## Methods

A COI model was developed from a service provider perspective of the UK NHS based on a pragmatic literature review and the opinion of clinical experts. The model used a prevalence-based approach to calculate the direct costs to the NHS and Personal Social Services of all patients diagnosed with CHI in the UK. Direct costs were defined as those arising from healthcare which was provided explicitly for, or because of, CHI.

### Pragmatic literature review

A treatment pathway specific to the UK context was developed following a pragmatic literature review, with electronic database searches conducted in MEDLINE via the PubMed platform on 4 September 2015. This was further supplemented with web-searching. The pathway was then refined in collaboration with clinical experts at GOSH [19] to identify four main treatment groups (Fig. 1): CHI patients treated at and discharged from their local hospital who did not undergo further tests (Group 1); CHI patients with focal disease (Group 2); CHI patients with diffuse disease – medically managed (Group 3); and CHI patients with diffuse disease – surgically managed (Group 4). The model included a consensus between the approaches of the two CHI specialist centres detailed above, as there was variability in the treatment approaches used. Both centres deemed the pathway to be in line with their standard practice at the time of model development (December 2015).

### COI model development

Model inputs were informed by the pragmatic literature review, including searches of NHS Reference Costs (2015–16), British National Formulary (BNF, 2017) and Office of National Statistics (ONS) National Life Tables (Mid-2015) [20–22]. All inputs were verified by clinical experts at GOSH and NORCHI [13, 19]. Key model inputs are listed in Table 1 (additional inputs are described in Additional file 1: Tables S1 to S3). Costs were calculated for individual hypothetical patients in each of the four treatment groups (as detailed above) based on the interventions, investigations and treatments that they would receive in accordance with the treatment pathway.

**Table 1:** Key inputs in the model

Input	Value	Reference
Population inputs		
Prevalence of CHI^b^	0.003%	Estimated scope of the national CHI service (NHS England CHI Service Standard Contract, 2013) [4]
Number of newly diagnosed patients with CHI per year^a,b^	95	Expert opinion (GOSH, 2015; NORCHI, 2016) [19, 30]
Maximum age of patients that have received a near-total pancreatectomy^b^	54	Harold N. Lovvorn III, 1999 [23]
Cost inputs per patient		
Daily cost of inpatient care at local hospital (excess)^a^	£486.62	NHS Reference Costs (2015–16) [20]
Daily cost of inpatient care at specialist centre (excess)^a^	£486.62	NHS Reference Costs (2015–16) [20]
Daily cost of neonatal care (excess)^a^	£274.11	NHS Reference Costs (2015–16) [20]
Cost of inpatient care, per non-elective long stay at local hospital^a^	£2186.61	NHS Reference Costs (2015–16) [20]
Annual cost of paediatric outpatient diabetes care^b^	£2925.00	NHS National Tariff (2014–15) [31]
Total annual cost of IDDM for patients managed by multiple daily insulin injections (including severe and non-severe events)^b^	£2183.00	Evans, 2015 (2013)* [32]
Cost of insertion of central venous catheter (Hickman line)^a^	£5925.98	NHS Reference Costs (2015–16) [20]
Clinical inputs		
Proportion of CHI patients responsive to first line therapy at local hospital^a,b^	75%	Expert opinion (NORCHI, 2016) [13]
Proportion of IDDM patients managed by multiple daily injections^b^	90%	Expert opinion (GOSH and NORCHI, 2016) [13, 19]
Incidence of IDDM at 11 years post near-total pancreatectomy^b^	96%	Arya, 2014 [15]
Proportion of CHI patients with diffuse disease responsive to octreotide^b^	30%	Expert opinion (NORCHI, 2016) [13]
Number of days until discharge from hospital following successful first-line therapy^a,b^	14	Expert opinion (GOSH and NORCHI, 2016) [13, 19]
Number of days to assess response to medical treatment in neonatal care^a^	10	Expert opinion (GOSH, 2016) [19]
Number of days from octreotide non-response until surgery^a^	21	Expert opinion (NORCHI), 2016 [30]

*Cost inflated to 2015/2016 cost year using the Personal Social Services Research Unit’s (PSSRU) hospital and community health services (HCHS) index [33]

Key inputs shown include the ten greatest cost drivers of first year costs^a^ and the ten greatest cost drivers of total annual costs^b^ as identified by the DSA

Assumptions made in the event of an absence of robust evidence or clinical data were validated by clinical experts at each treatment centre. Feedback from these experts was obtained via face-to-face meetings and questionnaire completion. Expert opinion from each centre was collected and averaged in order to derive a uniform clinical pathway representative of both centres. CHI and its associated treatments were assumed to have no effect on mortality: a mortality rate of 0% was applied to all patients and procedures.

Data from the ONS national life tables (2017) were used to calculate the number of CHI patients based on prevalence and incidence estimates (Table 1). The number of patients at each stage of the treatment pathway were estimated and multiplied by individual “per patient” costs to give the total COI. In the case of diffuse CHI patients that were surgically managed (assuming treatment would have been received in the first year of life), only patients born between 1963 and 2017 were considered [23]. This was due to the fact that the first case report of near-total pancreatectomy was published in 1963. CHI patients with diffuse disease born before 1963 (ie, exceeding the age limit the year the model was finalised, 2017) were assumed to have been unable to access treatment, leading to patient death.

Additional costs in the model included those associated with cognitive assessments for patients with CHI-related brain damage; however, long-term costs related to the additional medical and social care required by patients with neurodevelopmental delay were not included. Medical costs associated with IDDM onset following a near-total pancreatectomy were also included; these involved the costs of insulin and needles, as well as severe and non-severe hypoglycaemia events. However, these did not include costs attributed to secondary complications of diabetes such as heart disease, stroke, and nerve damage [16].

### Deterministic sensitivity analysis

A univariate deterministic sensitivity analysis (DSA) was run to determine the major first year and annual cost drivers. The DSA assessed the impact of variation in all parameters, with upper and lower estimates representing the impact on the final COI of increasing or decreasing the variable by 10%, respectively. The parameters were then ranked to determine which had the largest impact on costs when varied.

## Results

### Cost of CHI

**Fig. 2:**
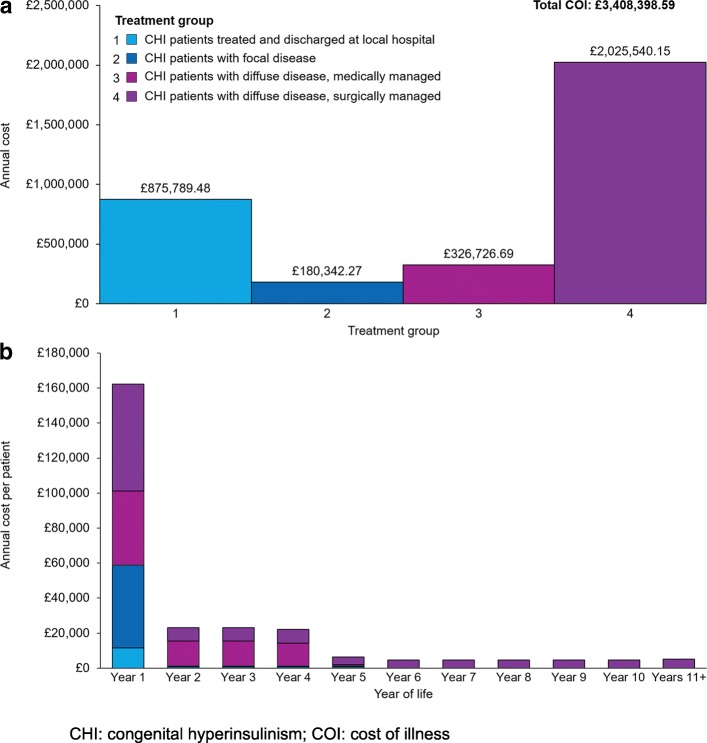
**a** Annual costs of CHI patients by treatment group and **b** annual per patient costs

The average annual cost of CHI to the NHS per patient per year was £2124.95 and the total annual COI to the NHS was £3,408,398.59 per year. CHI patients with diffuse disease that were surgically managed incurred the greatest total annual costs compared with other treatment groups. Total annual costs attributable to this treatment group were £2,025,540.15, representing 59% of the total cost of CHI to the NHS per year (Fig. 2a). This was the only treatment group to incur treatment costs after Year 5, with patients 10 years and older contributing to 48% of the total annual costs.

**Fig. 3:**
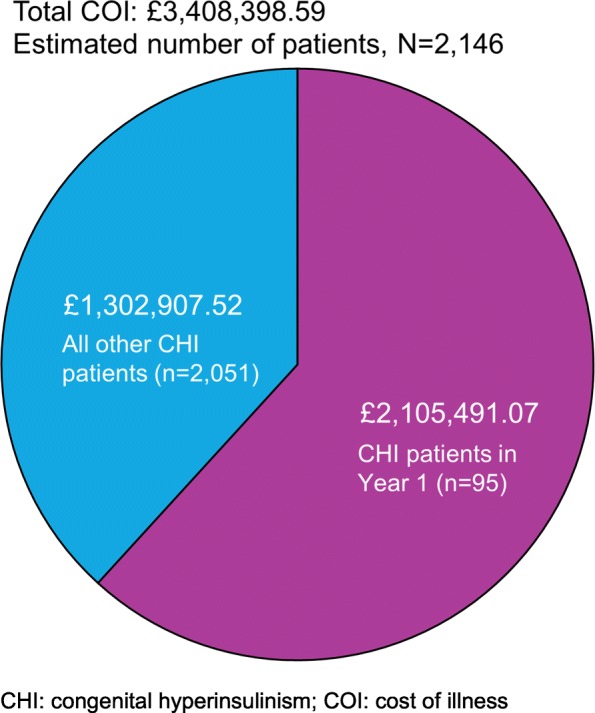
Estimated patient numbers and total costs of CHI patients (Year 1 and all other years)

Patients in their first year of life also represented a disproportionate share of total annual costs compared with other age groups (Fig. 3). Of the 2146 CHI patients in the UK, only 95 (4.4%) were in their first year of life. However, these patients incurred £2,105,491.07 in costs, representing 61.8% of the total cost of CHI to the NHS. Closer examination of the costs occurring within the first year of life showed that costs were evenly distributed between patients who were diagnosed, treated and discharged at a local hospital and those who were treated with a near-total pancreatectomy (39% [£819,408.55] and 41% [£853,894.45] of the costs attributable to this age group, respectively). CHI patients with diffuse disease treated with a near-total pancreatectomy also incurred the greatest per patient cost in the first year of life (Year 1). The annual cost of a single patient in each treatment group, stratified by age, is shown in Fig. 2b. Per patient costs were highest in Year 1 for all treatment groups and declined over time.

### Deterministic sensitivity analysis

**Fig. 4:**
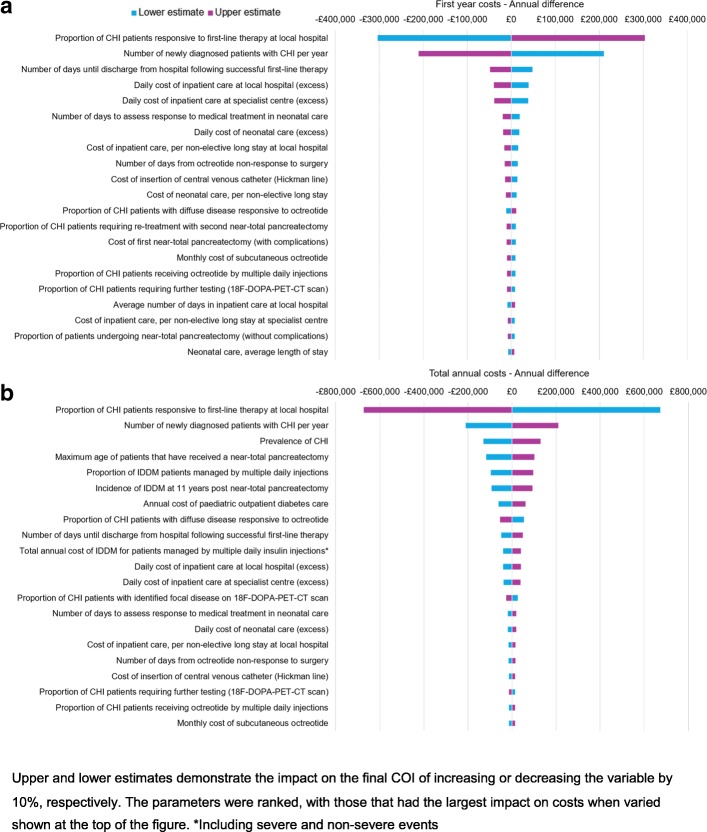
Tornado plots showing greatest cost drivers identified by the DSA

Tornado diagrams demonstrate the costs obtained when each parameter in the model was varied by 10%; lower and upper values are shown for both first year of life and annual costs (Fig. 4).

The DSA identified the proportion of patients unresponsive to first-line drug therapy to be the greatest driver of both first year of life and total annual costs. A 10% change in this parameter was estimated to alter total annual costs by £672,203.79. This is more than three times the £210,549.11 change produced by a 10% change in the number of newly diagnosed patients with CHI per year (the second greatest driver of total annual costs). The third and fourth biggest drivers of total annual costs were the prevalence of CHI and the maximum age of patients who have received a near-total pancreatectomy, respectively.

The number of newly diagnosed CHI patients per year was the second greatest driver of first year costs, followed by the number of days until discharge from hospital after successful first-line therapy.

## Discussion

Since the early 2000s, there has been a considerable increase in funding for research and development of rare disease treatments, likely aided by regulatory frameworks such as the Orphan Medicinal Products Regulation (2000) in the European Union [24]. Despite this impetus to develop new treatments for rare conditions, there continues to be a lack of robust clinical, economic and epidemiological data for the majority of rare diseases. This limits our understanding of the existing unmet need, and therefore the potential real-world impact of new treatments, including their anticipated effect on healthcare budgets.

CHI is no exception in terms of the lack of existing data in the literature. Subsequently, this COI model provides a much-needed estimate of the economic burden of CHI in the UK, and reports the treatment pathway followed by the majority of CHI patients. Data from a variety of sources have been used, including service providers, clinical experts and national statistics.

**Fig. 5:**
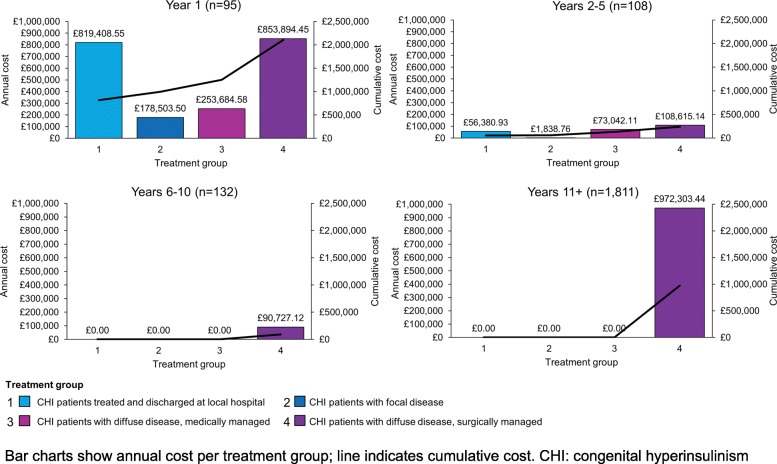
Annual costs grouped by year of life and stratified by treatment group

The results of this model indicate that patients in their first year of life incurred the greatest cost to the NHS compared to other age groups, and that management of CHI patients with diffuse disease requiring surgery accounts for a large proportion of these costs. CHI patients undergoing a near-total pancreatectomy in particular are a key driver of these high costs, due to the expensive nature of this procedure and associated costs such as extended hospital stays (relevant inputs shown in Additional file 1: Table S2 and S3). For patients in their first year of life (*n* = 95), costs were evenly distributed between patients who were diagnosed, treated and discharged at a local hospital and those who were treated with a near-total pancreatectomy, even though substantially fewer patients received this procedure (14/95 [15%]), than were treated at a local hospital (71/95 [75%]; Fig. 5). In addition, patients in their first year of life often receive multiple treatments as well as numerous tests and scans as they progress along the treatment pathway, incurring much higher costs than during maintenance therapy later in life.

As would be expected based on these results, non-response to first-line therapy was the greatest driver of total annual costs in the DSA. Patients responsive to first-line therapy undergo fewer treatments and do not undergo the genetic tests and scans, further medical therapy and/or near-total pancreatectomies experienced in other treatment groups.

Management of CHI patients with diffuse disease requiring surgery also incurred the greatest total annual cost compared with other treatment groups. This was due in part to the expensive nature of the required treatment, but was primarily driven by continuing care of patients aged 10 years and older, which accounted for 48% of the total annual cost of this treatment group. In addition, this major surgery is not always a ‘one-off’ procedure: based on expert opinion, our model assumed that 45% of patients who receive a near-total pancreatectomy would require a second procedure. [Expert opinion, GOSH and NORCHI] Initial excisions may not be sufficient, and patients could require implantation of feeding tubes.

Furthermore, the development of IDDM post surgery causes a large proportion of patients to require a lifetime of treatment for this condition, which represented the greatest of the costs included in the model. In addition to the trauma experienced by an infant undergoing invasive surgery, patients must also contend with the associated secondary complications of diabetes (increased risk of heart disease, stroke, nerve damage, diabetic retinopathy and chronic kidney disease [16]), as well as reduced quality of life, [25] and an increased risk of early mortality [26]. These secondary complications were not accounted for in this model and it is therefore likely that the total annual costs associated with this patient group are considerably higher than estimated.

In addition, the costs associated with comorbidities other than IDDM, especially those from brain damage acquired during prolonged hypoglycaemic episodes, were not fully captured by the model. Children who experience CHI-related brain damage may require long-term NHS funding for neurological morbidity e.g. costs for community-based occupational therapy and special educational needs. Such costs are substantial and dependent on the degree and duration of morbidity. In addition, families may bear additional costs, such as the cost of nappies for older children with incontinence caused by brain damage. Some families may also pay directly for further therapy, such as osteopathy, horse riding, and music therapy for their children [27]. Service provider costs associated with the long-term care of patients with brain damage were not included in the model as there is a high level of variability in neurodevelopmental outcomes; the treatment these patients receive cannot be easily generalised as care is personalised to individual outcomes. Subsequently, it is likely that the total economic burden is higher than suggested in this report. In addition, social care costs were not included as this model was developed using a service provider perspective. Inclusion of additional costs such as those associated with long-term support for patients with brain damage and secondary complications of IDDM would demonstrate the broader impact of CHI on the service provider over a patient’s lifetime.

Additional limitations of this model include the general lack of published data which, although expected when investigating a rare disease such as CHI, restricted the number of published sources from which to derive and verify inputs into the model. The model was instead developed using a consensus between the approaches of the only two centres of excellence in CHI in the UK, both based in England (London and Manchester/Liverpool). Subsequently, the UK-specific nature of the information collected may limit the generalisability of the model outside of the UK, given that treatment pathways are likely to vary internationally. Centralised treatment locations may allow for concentration of treatment efforts, through focused application of relevant expertise and maximal exposure of the healthcare professionals to CHI patients. This may help to standardise treatment strategies and improve the consistency of healthcare provision for patients.

The model considers a service provider perspective, and therefore only includes the direct costs of CHI to the NHS and Personal Social Services. While these costs are clearly substantial, the analysis did not account for the considerable economic burden experienced by families and caregivers of affected patients. Establishing an effective treatment regimen for an infant with CHI can be a time-consuming process involving frequent hospital visits over a period of months. Frequent travel to hospital as a result incurs significant out-of-pocket expenditure for parents and carers, and they may also suffer from serious losses of earnings and lack of promotion opportunities due to taking regular leave from work [Personal communication with Mr. R Thompson, 2017]. Families travelling long distances to reach GOSH or NORCHI, particularly those from Wales, Northern Ireland and Scotland, are particularly affected by this and may incur significant travel, accommodation and childcare costs. GOSH only provides accommodation for one parent at the hospital, and therefore the second parent must find alternative accommodation. Families with multiple children may also face substantial childcare costs. Inclusion of these additional costs would give a clearer understanding of the societal impact of CHI and therefore, further investigation into these implications is recommended. Future management strategies should seek to alleviate the burden experienced by patients’ families and caregivers while maximising quality of life for the individual.

Sirolimus is considered to be a new treatment option for CHI patients in the first year of life; it acts as an alternative to near-total pancreatectomy for CHI patients with diffuse disease, thereby allowing patients to potentially avoid subsequent onset of IDDM [2]. In a case report of four CHI patients with diffuse disease who were unresponsive to maximal doses of diazoxide and octreotide, treatment with sirolimus resulted in good glycaemic responses in all patients [2]. After the initiation of sirolimus, patients were soon able to maintain stable blood glucose levels without intravenous glucose infusions, which were gradually discontinued until patients were able to receive all nutrition enterally [2]. However, in a further study only 3 of 10 patients treated with mTOR inhibitors showed good glycaemic responses. This may have been due to a lack of association between mTOR signaling and disease in these patients, thus highlighting the importance of investigations into the identification of patient subgroups who are more likely to respond to new treatments, and the need for funding in clinician-led investigations [12]. In order to enable development of such treatments for rare disease patients, a large compilation of evidence is needed, which requires substantial time and funding. It is therefore essential that we continue to fund detailed clinical research to improve treatment availability.

In addition to prioritising treatment options such as sirolimus, other cost drivers which can be practically addressed include those related to length of stay, such as number of days until discharge from hospital following successful first-line therapy, number of days from octreotide non-response to surgery, and average number of days in inpatient care at local hospital (all of which were in the top 20 cost drivers of first year of life costs).

The results of this model have several potential policy implications. Both the UK Strategy for Rare Diseases and the EU-supported RARE-Best practices programme have indicated the value in undertaking research to address the gaps in knowledge and to define the best care pathways for rare diseases [28, 29]. This includes commitments from the UK Department of Health to “ensure that there are appropriate procedures for evaluating the costs and benefits of treatments for patients.” [28] Results from health economic studies such as this can inform evidence-based policies, and as a result, help to ensure that patients across the UK receive the same standard of care.

As the first ever COI model developed for CHI from the UK perspective, this model represents a crucial step forward in our understanding of the cost of this disease to the NHS. Through clinical expert validation, the model accurately reflects the full treatment pathway followed by these patients. The ability of the model to distinguish the costs for each age group enables a detailed view of the cost of this illness, and provides a novel tool to aid in identifying potential areas of cost savings.

## Conclusion

This study – which, to our knowledge is the first COI model published for CHI – found that despite being a rare disease, CHI is associated with substantial annual costs to the NHS and Personal Social Services. These findings add crucial information to a limited evidence base, and the identification of the management of post-surgical IDDM as a major cost driver highlights the need for effective treatments that could potentially reduce the number of patients experiencing the costly consequences of near-total pancreatectomy.

## Additional file

Additional file 1: **Table S1.** Population inputs in the model. **Table S2.** Cost inputs in the model. **Table S3.** Clinical inputs in the model. (DOCX 136 kb)

## Abbreviations

BNF: British National Formulary
CHI: Congenital hyperinsulinism
COI: Cost of illness
DSA: Deterministic sensitivity analysis
GOSH: Great Ormond Street Hospital
IDDM: Insulin-dependent diabetes mellitus
mTOR: Mechanistic target of rapamycin
NHS: National Health Service
NORCHI: Northern Congenital Hyperinsulinism Service
ONS: Office for National Statistics
