# Supplementary material for: The burden of congenital hyperinsulinism in the United Kingdom: a cost of illness study
Source: Orphanet J Rare Dis. 2018 Jul 20;13:123. doi: 10.1186/s13023-018-0867-6 (PMC6054726; doi:10.1186/s13023-018-0867-6)
Supplement: Supplementary file 1 — Table S1. Population inputs in the model. Table S2. Cost inputs in the model. Table S3. Clinical inputs in the model. (DOCX 136 kb) [file 13023_2018_867_MOESM1_ESM.docx]

Additional file 1

Table S1. Population inputs in the model

| Population inputs | Value | Reference |
| --- | --- | --- |
| General population, UK | 65,110,034 | Office for National Statistics, 2015[[1](#_ENREF_1)] |
| Prevalence of CHI | 0.003% | Estimated scope of the national CHI service (NHS England CHI Service Standard Contract, 2013)[[2](#_ENREF_2)] |
| Number of newly diagnosed patients with CHI per year | 95 | Expert opinion, (GOSH, 2015; NORCHI, 2016)[[3](#_ENREF_3),[4](#_ENREF_4)] |
| Maximum age of patients that have received a near-total pancreatectomy | 54 | Harold N. Lovvorn III, 1999 [[5](#_ENREF_5)] |

Table S2. Cost inputs in the model

| Cost inputs | Value | Reference |
| --- | --- | --- |
| Neonatal care | | |
| Cost of neonatal care, per non-elective long stay | £1,292.69 | NHS Reference Costs 2015-16[[6](#_ENREF_6)] |
| Daily cost of neonatal care (excess) | £274.11 | NHS Reference Costs 2015-16[[6](#_ENREF_6)] |
| CHI patients treated and discharged at local hospital | | |
| Cost of inpatient care, per non-elective long stay at local hospital | £2,186.61 | NHS Reference Costs 2015-16[[6](#_ENREF_6)] |
| Daily cost of inpatient care at local hospital (excess) | £486.62 | NHS Reference Costs 2015-16[[6](#_ENREF_6)] ^a^ |
| CHI patients who are transferred to specialist centre for treatment | | |
| Cost of transfer from local hospital to specialist centre, per journey | £990.19 | NHS Reference Costs 2015-16[[6](#_ENREF_6)] |
| Cost of insertion of central venous catheter (Hickman line) | £5,925.98 | NHS Reference Costs 2015-16[[6](#_ENREF_6)] |
| Cost of genetic testing (KATP analysis) | £1,000.00 | UK Genetic Testing Network 2017[[7](#_ENREF_7)] |
| Cost of inpatient care, per non-elective long stay at specialist centre | £2,186.61 | NHS Reference Costs 2015-16[[6](#_ENREF_6)] |
| Daily cost of inpatient care at specialist centre (excess) | £486.62 | NHS Reference Costs 2015-16[[6](#_ENREF_6)] ^a^ |
| Cost of a multidisciplinary team meeting to discuss genetic testing (KATP) results | £107.35 | NHS Reference Costs 2015-16[[6](#_ENREF_6)] ^b^ |
| Cost of ^18^F-DOPA-PET-CT scan, direct access | £2,149.97 | NHS Reference Costs 2015-16[[6](#_ENREF_6)] |
| Cost of ^18^F-DOPA-PET-CT scan, outpatient procedure | £544.63 | NHS Reference Costs 2015-16[[6](#_ENREF_6)] |
| Cost of transfer from specialist centre to ^18^F-DOPA-PET-CT scan, per journey | £236.44 | NHS Reference Costs 2015-16[[6](#_ENREF_6)] |
| CHI patients with focal disease | | |
| Daily cost of inpatient care at specialist centre | £486.62 | NHS Reference Costs 2015-16[[6](#_ENREF_6)] ^a^ |
| Cost of a multidisciplinary team meeting to discuss scan results and surgical approach | £107.35 | NHS Reference Costs 2015-16[[6](#_ENREF_6)] ^b^ |
| Cost of a laparoscopic lesionectomy | £3,234.15 | NHS Reference Costs 2015-16[[6](#_ENREF_6)] |
| Cost of an open lesionectomy | £8,117.84 | NHS Reference Costs 2015-16[[6](#_ENREF_6)] |
| Daily cost of bed days for laparoscopic lesionectomy (excess) | £380.64 | NHS Reference Costs 2015-16[[6](#_ENREF_6)] |
| Daily cost of bed days for open lesionectomy (excess) | £352.48 | NHS Reference Costs 2015-16[[6](#_ENREF_6)] |
| Cost of removal of central venous catheter (Hickman line), day case | £1,162.62 | NHS Reference Costs 2015-16[[6](#_ENREF_6)] |
| Cost of a multidisciplinary team meeting to discuss discharge from inpatient care | £107.35 | NHS Reference Costs 2015-16[[6](#_ENREF_6)] ^b^ |
| CHI patients with diffuse disease, medically managed | | |
| Daily cost of inpatient care at specialist centre | £486.62 | NHS Reference Costs 2015-16[[6](#_ENREF_6)] ^a^ |
| Daily cost of subcutaneous octreotide | £27.50 | Expert opinion (GOSH), 2015^*^ [[3](#_ENREF_3)] |
| Cost of gastrostomy insertion | £1,074.43 | NHS Reference Costs 2015-16[[6](#_ENREF_6)] |
| Cost of removal of central venous catheter (Hickman line), day case | £1,162.62 | NHS Reference Costs 2015-16[[6](#_ENREF_6)] |
| Cost of a multidisciplinary meeting to discuss discharge from inpatient care | £107.35 | NHS Reference Costs 2015-16[[6](#_ENREF_6)] ^b^ |
| CHI patients with diffuse disease, surgically managed | | |
| Daily cost of inpatient care at specialist centre (excess) | £486.62 | NHS Reference Costs 2015-16[[6](#_ENREF_6)] ^a^ |
| Daily cost of subcutaneous octreotide | £27.50 | Expert opinion (GOSH), 2015^* [^[^3^](#_ENREF_3)^]^ |
| Cost of a multidisciplinary team meeting to discuss scan results and surgical approach | £107.35 | NHS Reference Costs 2015-16[[6](#_ENREF_6)] ^b^ |
| Cost of first near-total pancreatectomy (without complications) | £6,737.17 | NHS Reference Costs 2015-16[[6](#_ENREF_6)] |
| Cost of first near-total pancreatectomy (with intraoperative and nonbiliary postoperative complications) | £9,225.62 | NHS Reference Costs 2015-16[[6](#_ENREF_6)] ^c^ |
| Cost of second near-total pancreatectomy (with complications) | £9,225.62 | NHS Reference Costs 2015-16[[6](#_ENREF_6)] ^c^ |
| Cost of choledochoduodenostomy | £3,881.93 | NHS Reference Costs 2015-16[[6](#_ENREF_6)] |
| Daily cost of bed days for near-total pancreatectomy, no complications (excess) | £396.31 | NHS Reference Costs 2015-16[[6](#_ENREF_6)] |
| Daily cost of bed days for near-total pancreatectomy, complications (intraoperative and nonbiliary postoperative) (excess) | £298.93 | NHS Reference Costs 2015-16[[6](#_ENREF_6)] |
| Daily cost of bed days for re-treatment with second near-total pancreatectomy (excess) | £298.93 | NHS Reference Costs 2015-16[[6](#_ENREF_6)] |
| Cost of closure of gastrostomy | £860.11 | NHS Reference Costs 2015-16[[6](#_ENREF_6)] |
| Cost of removal of central venous catheter (Hickman line), day case | £1,162.62 | NHS Reference Costs 2015-16[[6](#_ENREF_6)] |
| Cost of a multidisciplinary team meeting to discuss discharge from inpatient care | £107.35 | NHS Reference Costs 2015-16[[6](#_ENREF_6)] ^b^ |
| Post-discharge, CHI patients treated and discharged at local hospital | | |
| Cost of diazoxide (Eudemine^®^) per 50 mg tablet | £0.46 | BNF (February 2017)[[8](#_ENREF_8)] |
| Post-discharge, CHI patients with diffuse disease, medically managed | | |
| Daily cost of subcutaneous octreotide | £770.11 | Expert opinion (GOSH), 2015^*^ [[3](#_ENREF_3)] |
| Cost of lanreotide (Somatuline Autogel^®^) per 120 mg/mL pre-filled syringe | £551.00 | BNF (February 2017); Expert opinion (GOSH), 2015[[3](#_ENREF_3),[8](#_ENREF_8)] |
| Yearly cost of needles for multiple daily injections | £35.38 | Cummins, 2010 [Cost year NS]^*^ [[9](#_ENREF_9)] |
| Cost of insulin pump, per device | £2,626.12 | Cummins, 2010 [Cost year NS]^*^ [[9](#_ENREF_9)] |
| Yearly cost of infusion sets, reservoirs | £1,342.36 | Cummins, 2010 [Cost year NS]^*^ [[9](#_ENREF_9)] |
| Cost of a self-measured blood glucose test | £0.34 | Evans, 2015 [Cost year 2013]^*^ [[10](#_ENREF_10)] |
| Cost of a gastrostomy pump, for continuous feeds | £774.74 | Fresenius Kabi Price List, March 2017[[11](#_ENREF_11)] |
| Cost of closure of gastrostomy | £860.11 | NHS Reference Costs 2015-16[[6](#_ENREF_6)] |
| Cost of a gallbladder ultrasound scan | £55.14 | NHS Reference Costs 2015-16[[6](#_ENREF_6)] |
| Cost of gallbladder pathology treatment, ursodeoxycholic acid, per 60 x 150 mg tablets | £14.49 | BNF (February 2017)[[8](#_ENREF_8)] |
| Daily cost of inpatient care | £808.99 | NHS Reference Costs 2015-16[[6](#_ENREF_6)] |
| Post-discharge, CHI patients with diffuse disease, surgically managed | | |
| Cost of gastrostomy pump, for continuous feeds | £774.74 | Fresenius Kabi Price List, March 2017[[11](#_ENREF_11)] |
| Cost of closure of gastrostomy | £860.11 | NHS Reference Costs 2015-16[[6](#_ENREF_6)] |
| Cost of pancreatic exocrine assessment, sample taken at outpatient follow-up | £7.63 | NHS Reference Costs 2015-16[[6](#_ENREF_6)] |
| Cost of pancreatic enzyme replacement therapy, Pancrex^®^ V, per 300 capsules | £53.20 | BNF (February 2017)[[8](#_ENREF_8)] |
| Cost of pancreatic enzyme replacement therapy, CREON^®^ 25000, per 100 capsules | £28.25 | BNF (February 2017)[[8](#_ENREF_8)] |
| Post-discharge diabetes mellitus care for CHI patients with diffuse disease, surgically managed | | |
| Cost of paediatric outpatient diabetes care, per patient, per year | £2,925.00 | NHS National Tariff 2016-17[[12](#_ENREF_12)] |
| Total cost of insulin treatment for patients with IDDM managed by multiple daily insulin injections (including severe and non-severe events) per year | £2,183.00 | Evans, 2015 (2013)* [[10](#_ENREF_10)] |
| Average annual cost of insulin treatment with continuous subcutaneous insulin infusions | £2,858.87 | Cummins, 2010 [Cost year NS]^*^ [[9](#_ENREF_9)] |
| Cost of a hospital admission due to diabetes (excluding hypoglycaemic episode admission) | £2,136.83 | Cummins, 2010 [Cost year NS]^*^ [[9](#_ENREF_9)] |
| Cost of a hospital admission due to a hypoglycaemic episode | £837.04 | Cummins, 2010 [Cost year NS]^*^ [[9](#_ENREF_9)] |
| Follow-up visits for all patients | | |
| Cost of a follow-up outpatient visit | £228.68 | NHS Reference Costs 2015-16[[6](#_ENREF_6)] |
| Cost of annual cognitive assessment | £597.52 | NHS Reference Costs 2015-16[[6](#_ENREF_6)] |

*Cost inflated to 2015/2016 cost year using the Personal Social Services Research Unit’s (PSSRU) hospital and community health services (HCHS) index[[13](#_ENREF_13)]

^a^Cost input shares a cost reference described as “Paediatric Endocrine Disorders, excluding Diabetes Mellitus, with CC score 0”

^b^Cost input shares a cost reference described as “Other Cancer Multidisciplinary Team Meetings”

^c^Cost input shares a cost reference described as “Very Major Open, Hepatobiliary or Pancreatic Procedures, with CC Score 3”

Table S3. Clinical inputs in the model

| Clinical inputs | Value | Reference |
| --- | --- | --- |
| Neonatal care | | |
| Number of days to assess response to medical treatment in neonatal care | 10 | Expert opinion (GOSH), 2015[[3](#_ENREF_3)] |
| Proportion of CHI patients responsive to first-line therapy at local hospital | 75% | Expert opinion (NORCHI), 2016[[4](#_ENREF_4)] |
| Proportion of CHI patients requiring further investigation/treatment at specialist centre | 25% | Expert opinion (NORCHI), 2016[[4](#_ENREF_4)] |
| Average number of days in neonatal care | 2.79 | NHS Reference Costs 2015-16[[6](#_ENREF_6)] |
| CHI patients treated and discharged at local hospital | | |
| Number of days until discharge from hospital following successful first-line therapy | 14 | Expert opinion (GOSH, 2015; NORCHI, 2016)[[3](#_ENREF_3),[4](#_ENREF_4)] |
| Average number of days in inpatient care at local hospital | 2.59 | NHS Reference Costs 2015-16[[6](#_ENREF_6)] |
| CHI patients, transferred to specialist centre for treatment | | |
| Number of days until receive genetic testing result (KATP analysis) | 10 | Expert opinion (GOSH), 2015[[3](#_ENREF_3)] |
| Minimum number of days from receiving genetic testing result to ^18^F-DOPA-PET-CT scan | 22 | Expert opinion (GOSH), 2015[[3](#_ENREF_3)] |
| Proportion of CHI patients with genetically confirmed diffuse disease (GDH-HI, GK-H1) | 53% | Expert opinion (NORCHI), 2016[[4](#_ENREF_4)] |
| Proportion of CHI patients requiring further testing (^18^F-DOPA-PET-CT scan) | 47% | Expert opinion (NORCHI), 2016[[4](#_ENREF_4)] |
| Proportion of CHI patients with direct access to ^18^F-DOPA-PET-CT scan i.e. NORCHI | 47% | Expert opinion (GOSH, 2015; NORCHI, 2016)[[3](#_ENREF_3),[4](#_ENREF_4)] |
| Proportion of CHI patients without direct access to ^18^F-DOPA-PET-CT scan, i.e. GOSH | 54% | Expert opinion (GOSH, 2015; NORCHI, 2016)[[3](#_ENREF_3),[4](#_ENREF_4)] |
| Proportion of CHI patients with diffuse disease on ^18^F-DOPA-PET-CT scan | 66% | Expert opinion (NORCHI), 2016[[4](#_ENREF_4)] |
| Proportion of CHI patients with identified focal disease on ^18^F-DOPA-PET-CT scan | 34% | Expert opinion (NORCHI), 2016[[4](#_ENREF_4)] |
| Average number of days in inpatient care at specialist centre for insertion of central venous catheter (Hickman line) | 2.36 | NHS Reference Costs 2015-16[[6](#_ENREF_6)] |
| Average number of days in inpatient care at specialist centre | 2.59 | NHS Reference Costs 2015-16[[6](#_ENREF_6)] |
| CHI patients with focal disease | | |
| Number of days from focal disease diagnosis, i.e. scan results, to surgery | 17.5 | Expert opinion (GOSH), 2015[[3](#_ENREF_3)] |
| Number of days to discharge from laparoscopic lesionectomy | 7 | Expert opinion (NORCHI), 2016[[4](#_ENREF_4)] |
| Number of days to discharge from open lesionectomy | 10 | Expert opinion (NORCHI), 2016[[4](#_ENREF_4)] |
| Proportion of CHI patients undergoing laparoscopic lesionectomy | 40% | Expert opinion (NORCHI), 2016[[4](#_ENREF_4)] |
| Proportion of CHI patients undergoing open lesionectomy | 60% | Expert opinion (GOSH), 2015[[3](#_ENREF_3)] |
| Average number of days in hospital for a laparoscopic lesionectomy | 1.86 | NHS Reference Costs 2015-16[[6](#_ENREF_6)] |
| Average number of days in hospital stay for an open lesionectomy | 4.41 | NHS Reference Costs 2015-16[[6](#_ENREF_6)] |
| CHI patients with diffuse disease, medically managed | | |
| Number of days to assess response to octreotide | 8.5 | Expert opinion (GOSH), 2015[[3](#_ENREF_3)] |
| Number of days from assessment of response to octreotide to discharge | 1 | Expert opinion (GOSH), 2016[[14](#_ENREF_14)] |
| Proportion of CHI patients with diffuse disease responsive to octreotide | 30% | Expert opinion (NORCHI), 2016[[4](#_ENREF_4)] |
| Proportion of CHI patients with diffuse disease unresponsive to octreotide i.e. require near-total pancreatectomy | 70% | Expert opinion (NORCHI), 2016[[4](#_ENREF_4)] |
| Proportion of CHI patients responsive to octreotide requiring gastrostomy | 7.5% | Expert opinion (GOSH), 2016[[14](#_ENREF_14)] |
| Proportion of CHI patients not responsive to octreotide requiring gastrostomy | 15% | Expert opinion (GOSH), 2016[[14](#_ENREF_14)] |
| CHI patients with diffuse disease, surgically managed | | |
| Number of days from octreotide non-response until surgery | 21 | Expert opinion (NORCHI), 2016[[4](#_ENREF_4)] |
| Number of days on octreotide therapy until surgery | 19 | Arnoux, 2011[[15](#_ENREF_15)] |
| Number of days until discharge after a near-total pancreatectomy (without complications) | 10 | Expert opinion (GOSH), 2016[[14](#_ENREF_14)] |
| Number of days until discharge after a near-total pancreatectomy (with intraoperative and nonbiliary postoperative complications) | 21 | Expert opinion (GOSH), 2016[[14](#_ENREF_14)] |
| Number of days until discharge after a near-total pancreatectomy (with biliary postoperative complications) | 31.5 | Expert opinion (GOSH), 2016[[14](#_ENREF_14)] |
| Number of days from first surgery to second surgery | 17.5 | Expert opinion (GOSH), 2016[[14](#_ENREF_14)] |
| Number of days until discharge after re-treatment with a second near-total pancreatectomy | 31.5 | Expert opinion (GOSH), 2016[[14](#_ENREF_14)] |
| Proportion of CHI patients undergoing near-total pancreatectomy (without complications) | 54% | McAndrew, 2003[[16](#_ENREF_16)] |
| Proportion of CHI patients undergoing near-total pancreatectomy (with intraoperative and nonbiliary postoperative complications) | 35% | McAndrew, 2003[[16](#_ENREF_16)] |
| Proportion of CHI patients undergoing near-total pancreatectomy (with biliary postoperative complications) | 10% | McAndrew, 2003[[16](#_ENREF_16)] |
| Proportion of CHI patients still requiring gastrostomy post-near-total pancreatectomy | 33% | Cade, 1998[[17](#_ENREF_17)] |
| Proportion of CHI patients no longer requiring gastrostomy post-near-total pancreatectomy | 67% | Cade, 1998[[17](#_ENREF_17)] |
| Proportion of CHI patients requiring re-treatment with second near-total pancreatectomy | 45% | Expert opinion (GOSH and NORCHI), 2016[[4](#_ENREF_4),[14](#_ENREF_14)] |
| Average number of days in hospital for a near-total pancreatectomy (without complications) | 4.16 | NHS Reference Costs 2015-16[[6](#_ENREF_6)] |
| Average number of days in hospital for a near-total pancreatectomy (with intraoperative and nonbiliary complications) | 7.48 | NHS Reference Costs 2015-16[[6](#_ENREF_6)] |
| Average number of days in hospital for re-treatment with a second near-total pancreatectomy | 7.48 | NHS Reference Costs 2015-16[[6](#_ENREF_6)] |
| Average number of days in hospital for a choledochoduodenostomy | 2.27 | NHS Reference Costs 2015-16[[6](#_ENREF_6)] |
| Post-discharge, CHI patients treated and discharged at local hospital | | |
| Average weight of patient in year 1, kg | 7.11 | WHO, 2017[[18](#_ENREF_18)] |
| Average weight of patient, years 2–5, kg | 14.08 | WHO, 2017[[18](#_ENREF_18)] |
| Post-discharge, CHI patients with diffuse disease, medically managed | | |
| Average dose of octreotide, μg/kg/day | 17.8 | Demirbilek, 2014[[19](#_ENREF_19)] |
| Daily number of self-measured blood glucose tests for octreotide therapy | 7.5 | Expert opinion (GOSH), 2015[[3](#_ENREF_3)] |
| Annual number of inpatient bed days for octreotide therapy | 6 | Expert opinion (GOSH), 2015[[3](#_ENREF_3)] |
| Annual number of inpatient bed days for lanreotide therapy in the first and second year | 6 | Expert opinion (GOSH), 2015[[3](#_ENREF_3)] |
| Annual number of inpatient bed days for lanreotide therapy after the second year | 3 | Expert opinion (GOSH), 2015[[3](#_ENREF_3)] |
| Proportion of CHI patients receiving octreotide by continuous infusion | 0% | Expert opinion (GOSH), 2016[[14](#_ENREF_14)] |
| Proportion of CHI patients receiving octreotide by multiple daily injections | 100% | Expert opinion (GOSH), 2016[[14](#_ENREF_14)] |
| Proportion of CHI patients receiving bolus gastrostomy feeds only | 30% | Expert opinion (GOSH), 2016[[14](#_ENREF_14)] |
| Proportion of CHI patients receiving continuous gastrostomy feeds | 70% | Expert opinion (GOSH), 2016[[14](#_ENREF_14)] |
| Proportion of CHI patients switching from octreotide to lanreotide after first year | 50% | Expert opinion, (NORCHI), 2017[[20](#_ENREF_20)] |
| Proportion of CHI patients continuing with octreotide after first year | 50% | Expert opinion (NORCHI), 2017[[20](#_ENREF_20)] |
| Number of gallbladder ultrasound scans in the first year | 3 | Expert opinion (GOSH), 2015[[3](#_ENREF_3)] |
| Annual number of gallbladder ultrasound scans after the first year | 3 | Expert opinion (GOSH), 2015[[3](#_ENREF_3)] |
| Proportion of patients on octreotide developing gallbladder pathology | 32% | Demirbilek, 2014[[19](#_ENREF_19)] |
| Median weight of CHI patient at 3 months, kg | 6.40 | WHO, 2017[[18](#_ENREF_18)] |
| Median weight of CHI patient at 12 months, kg | 9.60 | WHO, 2017[[18](#_ENREF_18)] |
| Median weight of CHI patient at 24 months, kg | 12.20 | WHO, 2017[[18](#_ENREF_18)] |
| Post-discharge, CHI patients with diffuse disease, surgically managed | | |
| Proportion of CHI patients still requiring gastrostomy post-surgery | 33% | Cade, 1998[[17](#_ENREF_17)] |
| Proportion of CHI patients cured post-pancreatectomy | 18% | Arya, 2014[[21](#_ENREF_21)] |
| Proportion of CHI patients successfully managed with octreotide post-near-total pancreatectomy | 33% | Arya, 2014[[21](#_ENREF_21)] |
| Proportion of CHI patients successfully managed with diazoxide post-near-total pancreatectomy | 7% | Arya, 2014[[21](#_ENREF_21)] |
| Proportion of CHI patients successfully managed with regular daytime and overnight feeds post-near-total pancreatectomy | 16% | Arya, 2014[[21](#_ENREF_21)] |
| Proportion of CHI patients receiving bolus gastrostomy feeds only | 30% | Expert opinion (GOSH), 2016[[14](#_ENREF_14)] |
| Proportion of CHI patients receiving continuous gastrostomy feeds | 70% | Expert opinion (GOSH), 2016[[14](#_ENREF_14)] |
| Proportion of CHI patients requiring pancreatic enzyme supplementation post-surgery | 49% | Arya, 2014[[21](#_ENREF_21)] |
| Post-discharge diabetes mellitus care for CHI patients with diffuse disease, surgically managed | | |
| Proportion of CHI patients requiring transient insulin therapy immediately post-near-total pancreatectomy | 9% | Arya, 2014[[21](#_ENREF_21)] |
| Incidence of IDDM immediately post-near-total pancreatectomy | 0.13 | Arya, 2014[[21](#_ENREF_21)] |
| Incidence of IDDM at 1 year post-near-total pancreatectomy | 0.18 | Arya, 2014[[21](#_ENREF_21)] |
| Incidence of IDDM at 2 years post-near-total pancreatectomy | 0.04 | Arya, 2014[[21](#_ENREF_21)] |
| Incidence of IDDM at 3 years post-near-total pancreatectomy | 0.04 | Arya, 2014[[21](#_ENREF_21)] |
| Incidence of IDDM at 4 years post-near-total pancreatectomy | 0.09 | Arya, 2014[[21](#_ENREF_21)] |
| Incidence of IDDM at 5 years post-near-total pancreatectomy | 0.13 | Arya, 2014[[21](#_ENREF_21)] |
| Incidence of IDDM at 6 years post-near-total pancreatectomy | 0.09 | Arya, 2014[[21](#_ENREF_21)] |
| Incidence of IDDM at 7 years post-near-total pancreatectomy | 0.18 | Arya, 2014[[21](#_ENREF_21)] |
| Incidence of IDDM at 11 years post-near-total pancreatectomy | 0.96 | Arya, 2014[[21](#_ENREF_21)] |
| Proportion of CHI patients with IDDM managed by multiple daily injections | 90% | Expert opinion (GOSH, 2015; NORCHI, 2016)[[3](#_ENREF_3),[4](#_ENREF_4)] |
| Proportion of CHI patients managed by continuous subcutaneous insulin infusions | 10% | Expert opinion (GOSH, 2015; NORCHI, 2016)[[3](#_ENREF_3),[4](#_ENREF_4)] |
| Annual number of diabetes admissions (excluding hypoglycaemic episode admission) | 0 | Expert opinion (NORCHI), 2016[[4](#_ENREF_4)] |
| Annual incidence of severe hypoglycaemic events | 0.1150 | Cummins, 2010[[9](#_ENREF_9)] |
| Follow-up visits for all patients | | |
| Annual number of follow-up visits for CHI patients who are diagnosed and treated successfully with first-line therapy and discharged | 1 | Expert opinion (GOSH), 2015[[3](#_ENREF_3)] |
| Annual number of follow-up visits for CHI patients with focal disease, post-surgery | 1 | Expert opinion (GOSH), 2015[[3](#_ENREF_3)] |
| Annual number of follow-up visits for medically managed CHI patients with diffuse disease | 3 | Expert opinion (GOSH), 2015[[3](#_ENREF_3)] |
| Annual number of follow-up visits for surgically managed CHI patients with diffuse disease | 3 | Expert opinion (GOSH), 2015[[3](#_ENREF_3)] |
| Annual number of follow-up visits for CHI patients on sirolimus treatment | 3 | Expert opinion (GOSH), 2015[[3](#_ENREF_3)] |
| Proportion of CHI patients receiving cognitive assessments | 33% | Expert opinion (GOSH, 2015; NORCHI, 2016)[[3](#_ENREF_3),[4](#_ENREF_4)] |

SUPPLEMENTARY REFERENCES

1. Office of National Statistics. National Life Tables (Mid–2015). Available from: [www.ons.gov.uk/peoplepopulationandcommunity/birthsdeathsandmarriages/lifeexpectancies/datasets/nationallifetablesenglandandwalesreferencetables](http://www.ons.gov.uk/peoplepopulationandcommunity/birthsdeathsandmarriages/lifeexpectancies/datasets/nationallifetablesenglandandwalesreferencetables). [Accessed March 20, 2017].

2. NHS England. 2013/14 Standard Contract for Congenital Hyperinsulism Service (Children). Available from: [www.england.nhs.uk/wp-content/uploads/2013/06/a17-congen-hyper-ser-child.pdf](http://www.england.nhs.uk/wp-content/uploads/2013/06/a17-congen-hyper-ser-child.pdf) [Accessed March 21 2017].

3. Expert Opinion (GOSH). Expert Opinion Received from Great Ormond Street Hospital 2015.

4. Expert Opinion (NORCHI). Expert Opinion Received from the Northern Congenital Hyperinsulinism Service. 2016.

5. Lovvorn HN, III, Nance ML, Ferry RJ, Jr., et al. Congenital hyperinsulinism and the surgeon: Lessons learned over 35 years. *J Pediatr Surg.* 1999;34(5):786–793.

6. National Health Service. NHS Reference Costs 2015–16. Available from: [www.gov.uk/government/uploads/system/uploads/attachment_data/file/577084/National_schedule_of_reference_costs_-_main_schedule.xlsx](http://www.gov.uk/government/uploads/system/uploads/attachment_data/file/577084/National_schedule_of_reference_costs_-_main_schedule.xlsx). [Accessed March 3, 2017].

7. UK Genetic Testing Network. Hyperinsulinemic Hypoglycemia, Familial, 1. Available from: <https://ukgtn.nhs.uk/find-a-test/search-by-disorder-gene/hyperinsulinemic-hypoglycemia-familial-1-403>. [Accessed March 30, 2017].

8. Joint Formulary Committee. British National Formulary. Available from: <https://bnf.nice.org.uk/drug/>. [Accessed March 3, 2017].

9. Cummins E, Royle P, Snaith A, et al. Clinical effectiveness and cost-effectiveness of continuous subcutaneous insulin infusion for diabetes: systematic review and economic evaluation. *Health Technology Assessment.* 2010;14(11).

10. Evans M, Wolden M, Gundgaard J, Chubb B, Christensen T. Cost-effectiveness of insulin degludec compared with insulin glargine in a basal-bolus regimen in patients with type 1 diabetes mellitus in the UK. *J Med Econ.* 2015;18(1):56–68.

11. Fresenius Kabi. Fresenius Kabi Price List March 2017. Available from: [www.fresenius-kabi.co.uk/6893.htm](http://www.fresenius-kabi.co.uk/6893.htm). [Accessed March 21, 2017].

12. National Health Service. NHS National Tariff Payment System 2016/17. Available from: [www.gov.uk/government/uploads/system/uploads/attachment_data/file/509698/Annex_A_national_prices_and_national_tariff_workbook.xlsx](http://www.gov.uk/government/uploads/system/uploads/attachment_data/file/509698/Annex_A_national_prices_and_national_tariff_workbook.xlsx). [Accessed September 13, 2016].

13. Personal Social Services Research Unit. Unit Costs of Health and Social Care. Available from: [www.pssru.ac.uk/project-pages/unit-costs/2016/](http://www.pssru.ac.uk/project-pages/unit-costs/2016/). [Accessed March 21, 2017].

14. Expert Opinion (GOSH). Expert Opinion Received from Great Ormond Street Hospital 2016.

15. Arnoux JB, Verkarre V, Saint-Martin C, et al. Congenital hyperinsulinism: current trends in diagnosis and therapy. *Orphanet J Rare Dis.* 2011;6:63.

16. McAndrew HF, Smith V, Spitz L. Surgical complications of pancreatectomy for persistent hyperinsulinaemic hypoglycaemia of infancy. *J Pediatr Surg.* 2003;38(1):13–16.

17. Cade A, Walters M, Puntis JW, Arthur RJ, Stringer MD. Pancreatic exocrine and endocrine function after pancreatectomy for persistent hyperinsulinaemic hypoglycaemia of infancy. *Arch Dis Child.* 1998;79(5):435–439.

18. World Health Organization. Weight-for-age child growth standards birth to 5 years. Available from: [www.who.int/childgrowth/standards/weight_for_age/en/](http://www.who.int/childgrowth/standards/weight_for_age/en/). [Accessed March 3, 2017].

19. Demirbilek H, Shah P, Arya VB, et al. Long-term follow-up of children with congenital hyperinsulinism on octreotide therapy. *J Clin Endocrinol Metab.* 2014;99(10):3660–3667.

20. Expert Opinion (NORCHI). Expert Opinion Received from the Northern Congenital Hyperinsulinism Service. 2017.

21. Arya VB, Senniappan S, Demirbilek H, et al. Pancreatic endocrine and exocrine function in children following near-total pancreatectomy for diffuse congenital hyperinsulinism. *PLoS One.* 2014;9(5):e98054.
